# Feasibility Evaluation of Designated Quantities for Chemicals Requiring Preparation for Accidents in the Korean Chemical Accident Prevention System

**DOI:** 10.3390/ijerph17061927

**Published:** 2020-03-16

**Authors:** Mun Seob Ahn, Hyo Eun Lee, Kwang Soo Cheon, Huoung Gi Joo, Ochang Chemical Safety Community, Bu-Soon Son

**Affiliations:** 1 Samsung SDI Co. Ltd., 146, Gwahaksaneop 4-ro, Ochang-eup, Chongwon-gu, Chongju-si, Chungcheongbuk-do 28121, Korea; plussubi1982@naver.com (M.S.A.); hyounggi.joo@samsung.com (H.G.J.); 2 Department of Environmental Health Science, Soonchunhyang University, 22, Soonchunhyang-ro, Sinchang-myeon, Asan-si, Chungcheongnam-do 31538, Korea; 3 Department of Health Science, Korea University, Anam-ro 145, Seongbuk-gu, Seoul 02841, Korea; chokbab@naver.com; 4 Ministry of Environment, Chemical Safety Management Department, 417 Daehak-ro, Youseoung-Gu Daejeon 34142, Korea; chunks71@korea.kr; 5 Ochang Chemical Safety Community, 146, Gwahaksaneop 4-ro, Ochang-eup, Chongwon-gu, Chongju-si, Chungcheongbuk-do 28121, Korea; kimys@wonik.com

**Keywords:** quantity of chemicals requiring preparation, pool fire, vapor cloud explosion (VCE), Areal Location of Hazardous Atmosphere (ALOHA), Korea Off-Site Risk Assessment Supporting Tool (KORA), flammable substances, risk management plan (RMP), process safety management (PSM)

## Abstract

To prevent chemical accidents, the United States (US), the European Union (EU), and the Republic of Korea operate legal systems, such as risk management plans (RMP) and process safety management (PSM), to prevent chemical accidents inside and outside the workplace. The duty to implement chemical accident prevention systems and the criteria for being a target workplace are dependent on the designated quantities of chemicals handled. A chemical accident prevention system is obligatory for storage and handling of legally declared chemicals in the workplace. Benzene, toluene, xylene, methyl ethyl ketone, and ethyl acetate are all flammable materials that are commonly used as solvents in the chemical industry. These substances are grouped into flammable substances groups in the US and the EU, and are managed with the same designated quantities. However, in Korea, the designated quantities are: benzene, 10,000 kg; toluene, xylene, and methyl ethyl ketone, 200,000 kg; and ethyl acetate, 20,000 kg. In order to evaluate the validity of the chemical quantities, fire explosion scenarios during chemical accidents were modeled using two modeling programs, Areal Location of Hazardous Atmosphere (ALOHA) and Korea Off-Site Risk Assessment Supporting Tool (KORA) software, under the same conditions. Similar damage radii were found for the five flammable materials with both pool fires and vapor cloud explosions (VCE). Based on these damage radii, the designated quantities of five substances were calculated and included in the range (10,000 to 13,500 kg). The results show that current designated quantities underestimate chemical substances, and for the prevention of accidents and post-management after chemical accidents, it is necessary to manage flammable substances under one grouping.

## 1. Introduction

The chemical industry and related industries have been the driving forces behind the Republic of Korea’s development since 1960. In 1960, Korea’s per capita income was USD 82. At that time, India’s per capita income was USD 85. By developing a chemical-based industry and transitioning from the government-led light industry to the heavy and chemical industries, South Korea rose to a national income of USD 20,000 per capita in 2019 [1]. Economic development has enriched people’s lives and has made Korea one of the most advanced countries. However, the growth of the chemical industry has increased the likelihood of chemical accidents. Safety awareness is learned from accidents. For example, the 2012 Gumi hydrogen fluoride accident in South Korea resulted in the Chemicals Control Act. As a result, a process safety system was introduced [2].

Chemical accidents can be divided into those caused by toxic chemical leakage, fires, and explosions. The mechanisms of chemical accidents vary considerably, depending on whether the chemical itself is toxic or flammable. In the case of accidents caused by toxic substances, first response and post-treatment are important after spreading or leaking to the surrounding environment. However, if a fire or explosion occurs, the accident could potentially damage adjacent chemical facilities. Additionally, in the case of a leak that does not accompany a fire, liquid chemicals can minimize the damage by preventing the initial response and preventive action, however the leakage is difficult to cope with because fires and explosions can cause extensive damage very quickly [3].

In order to prevent various chemical accidents worldwide, a process safety management (PSM) and risk management plan (RMP) scheme is operated. This is a system of prevention and aftercare treatment at the level of management [4].

However, this does not apply to all chemicals, rather it only applies to the storage or handling of a certain quantity of declared chemicals (e.g., plants storing more than 10,000 kg of hydrochloric acid) [5].

Commonly used in various industries, chemicals that can cause great damage, such as fires and explosions in chemical accidents, are designated as “chemicals requiring preparation” in Korea. These substances are widely used in Korea and are composed of substances that cause great danger in the case of a chemical accident. In particular, toluene, xylene, ethyl acetate, methyl ethyl ketone, and benzene have five physicochemical properties and are mainly used as solvents in the chemical industry. These five substances are widely used worldwide and are included in each country’s safety management system [6,7].

The RMP system in Korea introduces the concept of designated quantities for “chemicals requiring preparation” and establishes an RMP plan when the maximum amount of stored chemicals is exceeded. In order to prevent accidents, it is easy to approach safety measures based on large storage facilities. In addition, regulations and process controls are diverse and difficult to apply. The management aspect of the workplace is also an important element in risk assessment, however this study focused on regulation of the total amount of chemicals, which is an aspect that is easy and important to manage in the workplace [8]. The same is true for the US and the EU. However in Korea, the designated quantity is determined by a combination of factors, such as chemical usage, toxicity, and physical properties. The designated quantity is a concept relating to how big a tank is in a factory. It is very likely that a fire or an explosion will occur in a storage tank, and even in the same “chemicals requiring preparation”, the specified quantities vary greatly (between 750 and 20,000 kg) [9].

In particular, the above-mentioned chemicals of benzene, toluene, xylene, ethyl acetate, and methyl ethyl ketone also differ in the designated quantities of each chemical, ranging from 10,000 to 200,000 kg. Despite similar physicochemical properties, the difference in designated quantities is more than 20 times the risk application in terms of chemical accident prevention [6,7]. Therefore, in this study, the modeling is based on the assumption that a chemical accident has occurred for these five substances, and compared with the specified quantity in other countries, the study intends to present the designated quantity of each chemical as conservatively as possible.

## 2. Materials and Methods

### 2.1. Selected Chemical Substances

In Korea, substances that have a high risk of accidents are used in general industries, while those that have high physicochemical properties, such as flammability, explosion potential, reactivity, and leakage potential, are designated as “chemicals requiring preparation”. As of 2020, 97 types of “chemicals requiring preparation” have been designated in Korea [10]. Workplaces handling more than a certain quantity of these chemicals should prepare a risk management plan (RMP) to establish their own accident prevention and response methods, and submit the plan to the Ministry of Environment’s Chemical Safety Agency. The summary of the RMP is obliged to inform local residents of the municipality and to protect local residents against chemical accidents [11].

There are two types of chemical accident prevention systems, PSM and RMP, which are also being implemented in the US and the EU. In this study, chemicals with flammable properties that could cause extensive damage from fires and explosions were selected among various chemicals. The selected materials are benzene, methyl ethyl ketone, toluene, xylene, and ethyl acetate. Table 1 compares the physical and chemical properties [12], as well as the specified quantities [5]. Physical and chemical properties related to chemical accidents, such as fires and explosions, of the material under investigation include the flash point, explosion upper and lower limits, and vapor pressure. It is evident that the physicochemical properties of the five chemicals are similar.

### 2.2. Comparison of Designated Quantity in Each Country’s Accident Prevention System

There are various systems in each country to prevent chemical accidents.

In the US, chemical accident prevention is promoted by the Environmental Protection Agency (EPA) and the Occupational Safety and Health Administration (OSHA). The EPA implements the RMP scheme in accordance with Federal Regulation 40CFR68, while the OSHA establishes the obligations of high-risk substance handling establishments in accordance with Federal Regulation 29CFR 1910,119. The purpose of each system is to cooperate with local governments in addition to local police and fire departments in the case of a chemical accident, and to disclose information to local residents. On the other hand, for PSM, the purpose is to protect workers in the workplace, for which in-process accident prevention management is the key. Both systems are subject to establishment plans for workplaces that handle chemicals in volumes exceeding the specified quantity.

In the EU, the United Kingdom (UK)’s Control of Major Accident Hazard (COMAH) is being implemented by applying the 2005 SEVESO II Directive. In other words, the EU has followed the SEVESO II Directive but recognizes autonomy by country. Within the COMAH legislation, when the workplace handles chemicals above the specified quantity, a process safety report must be submitted [13].

In Korea, similar to the US system, two departments, namely the Ministry of Environment and the Ministry of Employment and Labor, implement and administer a chemical accident prevention system. The Department of Environment introduced a RMP, which satisfies the local residents right-to-know, as well as prevents the spread of chemical accidents in collaboration with local governments. The PSM is administered by the Ministry of Employment and Labor, and is implemented for the purpose of establishing rules for the protection of workers’ health and the management of workplace processes. For each system applied in each country, Table 2 shows the comparison of the designated quantity, which is the legal standard for the study subject matter [5,6,7,14].

In the US and the UK, chemicals with a flash point below 60 °C are managed in the same way as flammable liquids; however, in Korea, specific quantities are specified for each chemical. The problem with the Korean system is that although the chemicals have similar physicochemical properties that are likely to cause chemical accidents, the specified quantities vary from 10,000 to 200,000 kg [15].

China, a neighboring country, has not yet implemented the PSM system. However, The Emergency Event Response Law was passed in 2007 in response to the gas well blowout in Chongqing, China (2003), and the petrochemical plant explosion and subsequent release of chemicals into the Songhua River in Jilin Provence, China (2005). This law requires chemical plants to create an emergency response plan for any potential incidents. These laws are being implemented and international safety systems are increasingly being introduced. India is making efforts, such as designing processes similar to OSHA PSM standards, however with no restrictions. Regulatory provisions in India include exposure limits for workers under The Factories Act [16].

### 2.3. Chemical Accident Case

An accident caused by a chemical is a combination of the properties of a chemical and the characteristics of the process in which the chemical is handled (e.g., operating and pressure conditions), along with the surrounding meteorological environment (e.g., temperature and humidity). There are three types of chemical accidents: toxic substance leakage, fire, and explosion. Fires include pool fires and jet fires, while explosions include vapor cloud explosions (VCE) and boiling liquid expanding vapor explosions (BLEVE) [3].

A pool fire is a fire that occurs when ignition occurs in an open tank, pool, or flowing liquid, with the liquid level exposed to the atmosphere. This is a type of fire that occurs when chemicals leaked from storage tanks accumulate in the discharge barrier and ignite due to an ignition source.

A jet fire refers to a fire that is ignited by a liquid chemical ejected from a pressurized chemical transfer pipe or a pressurized pump, or from a small leak from a high-pressure container. This fire type can be prevented, however is difficult to extinguish, because if it occurs in a tank it is difficult to access.

VCE is an explosion phenomenon caused by an ignition source after a large amount of flammable vapor or gas has been rapidly leaked into the atmosphere. It often occurs indoors, however if there is no wind blowing or there is a stable weather environment, it can also occur outdoors.

**Figure 1:**
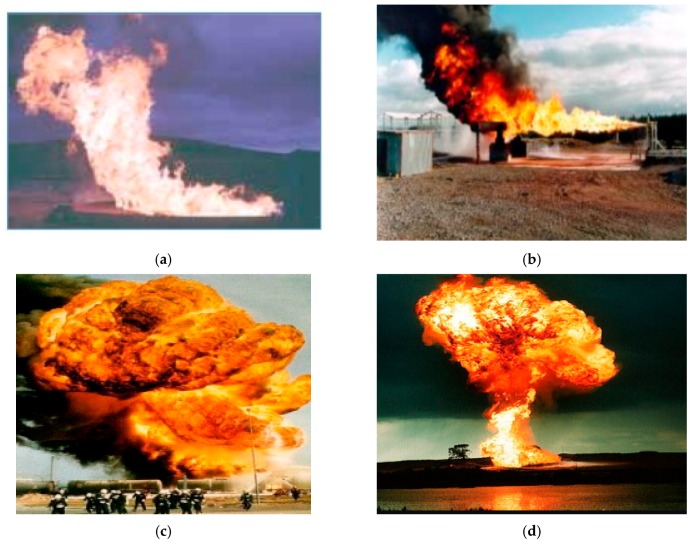
Example of each fire explosion case: (**a**) pool fire, (**b**) jet fire, (**c**) boiling liquid expanding vapor explosion (BLEVE), and (**d**) vapor cloud explosion (VCE).

BLEVE is an explosion phenomenon in which overheated and over pressurized liquid suddenly boils due to rupture of the container in high-pressure or closed equipment. A fire ball is formed after the explosion and the damage is extensive. These explosions occur in the gas storage tank, rather than with chemicals. A system is needed to cool the tank to prevent it from being heated by flames or radiant heat. Figure 1 shows an example of each kind of fire explosion [17].

Due to the physicochemical properties of the study materials, fire and explosion cases will cause more damage from chemical accidents than leakage of toxic substances. In addition, dual jet fires and BLEVEs are cases that can occur in high-pressure equipment. The designated quantity means the maximum storage volume, relating to storage tanks that are stored at atmospheric pressure. Therefore, in this study, we investigated VCE and pool fire scenarios, which are chemical accident cases that can occur at normal pressures.

#### 2.3.1. Vapor Cloud Explosion (VCE)

A vapor cloud explosion is an explosion that can occur when a flammable material is leaked and vaporized by vapor pressure. Three conditions must be met for this explosion to be true:

(1) The substance in the cloud must be within its flammability limits. These are temperature limits within which it is possible for a flammable substance to ignite, depending on the kind of substance;

(2) Ignition must be delayed. If the cloud starts burning before it is completely formed, other fire scenarios would occur instead of a VCE. Such other events are outside of the scope of this paper;

(3) Turbulence must be present in the cloud. The release mode of the substance (a jet, for example) can trigger this turbulence. Interaction with close objects may also cause partial confinement, generating turbulence within the cloud.

Under these conditions, once an explosion begins, the combustion energy is replaced with physical energy to create a flame. These flame storms generate high pressure and high heat, and continue until all steam is burned [18].

Analysis methods of vapor cloud explosions include the trinitrotoluene (TNT) equivalence approach method, the Netherlands Organization (TNO) multi-energy method, and the Baker–Strehlow–Tang (BST) method. The TNT equivalence approach is a method for calculating the overpressure, assuming that the flammable energy in the steam cloud is converted to a significant amount of TNT (1–10%), and is the most widely used method. However, it is not compatible with some flammable liquids, and there is a problem whereby explosion accidents caused by deflagration result in overestimation.

The TNO multi-energy method assumes that if the explosive energy depends on the degree of gas density in the combustible area, and if it occurs in confined spaces or obstacles exist, only the area occupied by the actual cloud will contribute to the storm. This model also has the concept of a single explosion, where the explosion starts in one place rather than a series of explosions in different parts of the steam cloud.

The Baker–Strehlow–Tang (BST) method is similar to the TNO multi-energy model. In particular, it is a model that assumes that an explosion starts in the part of a flammable vapor cloud where the process equipment is concentrated [19].

#### 2.3.2. Pool Fire

Pool fires are generally caused by the release of flammable chemicals in storage tanks. A lack of initial suppression creates the risk of the liquid chemicals continually increasing the area of the pool, increasing the risk of fire. In general, the heat release rate (HRR) in pool fires is a measure of how large a fire will be [20].

(1) $$Q^{˙}=x_{a}\times m^{\prime \prime }\times A_{s}\times \Delta H_{c}$$

$Q^{˙}$ = heat release rate (HRR), x_a_ = combustion efficiency, m^″^ = mass loss rate per unit area (MLRPUA), A_s_ = pool surface area, ΔH_c_ = heat of complete combustion, Equation (1) parameters for characterizing the fire hazards for pool fire.

### 2.4. Selection of Risk Assessment Conditions

The condition for risk assessment was calculated according to the designated quantity in each country’s accident prevention system. The current designated quantity means the maximum quantity that is stored at each workplace. Therefore, this was estimated for storage tanks among various chemical facilities, such as for storage, reaction, and mixing tanks.

In addition, the operating conditions are assumed to outdoors at room temperature and pressure. Currently, Korea’s designated quantities are different from each other, so it is assumed that 10,000 kg is stored based on the limit for benzene, the lowest standard among the five chemicals. This is a common reference value for the US and the EU. It is necessary to model all chemicals under the same conditions when assuming a chemical accident and then recalculate the designated quantity for each chemical according to the results.

Table 3 shows the basic specifications of storage tanks that can store each chemical.

The storage tanks also have a dike to prevent leakage to the outside. This dike is an important factor in calculating pool fires. The standard size of a dike wall was calculated as 110% of the storage tank’s capacity, which is currently required to comply with the Chemicals Control Act and the US Dangerous Goods Control Act [11].

Important meteorological conditions in the modeling were applied with reference to the selection guidelines for chemical accident modeling published by the US EPA, as well as the modeling guidelines published by the Korea Chemical Safety Agency [21,22]. Table 4 defines the Pasquill atmospheric stability.

### 2.5. Selection of Tool for Chemical Accident Risk Assessment

There are many ways to predict damage by modeling chemical accidents. Currently, many tools are used to predict chemical accidents. A well-known program is the Areal Location of Hazardous Atmosphere (ALOHA) software, developed by the US EPA. ALOHA applies the Baker–Strehlow–Tang (BST) method as a VCE modeling technique. There is also the Korea Off-Site Risk Assessment Supporting Tool (KORA) software, a nationally developed program with the introduction of the Chemicals Control Act in Korea. This program supports the TNO method for the pool fire and VCE models. In this study, the modeling was carried out using the TNO method, which in the other previous studies was predicted to cause significant damage, to proceed in a more conservative manner [19]. The TNT model mentioned in this study was excluded because it was not suitable for flammable liquids. These two programs, unlike other modeling programs, have the advantage that they can be used free of charge in all countries. In particular, in Korea and in other countries, they can be applied as tools to be used for future risk assessment and chemical response legislation.

Table 5 shows the characteristics, advantages, and disadvantages of the two modeling programs and the behavior analysis theory of the VCE model.

## 3. Results

### 3.1. Background

There are three main characteristics of chemicals in determining risk. These are the physical hazards, health hazards, and environmental hazards. Physical hazards are explosive, oxidizing, and flammable materials. Acute toxicity, skin corrosion, and irritation are typical in health hazards. Reproductive toxicity and aquatic environments are typical characteristics in environmental hazards. As an example of how widespread chemical usage is [23], in 2015, about 150,000 chemicals were distributed worldwide, and about 2000 new chemicals enter the market every year. The Organization for Economic Cooperation and Development (OECD) forecasts that by 2020, the production of chemicals will increase by more than 80% compared with 1995, and this is becoming a reality. Continued increases in the use of these chemicals will increase the risk of exposure to people and ecosystems and the likelihood of chemical accidents. As a result, international regulations need to be strengthened, and international information exchange and cooperation for chemicals has becomes more important. Therefore, the United Nations Environment Program (UNEP) adopted the globally harmonized system (GHS) to apply a classification system for chemicals around the world [24].

In developed countries, such as the United States and European countries, since the 1970s, due to the occurrence of chemical accidents, GHS classification has established a policy to prevent chemical accidents based on physical hazard, among various characteristics. However, in 2003, the GHS system was introduced in Korea, and regulations for health and environmental hazards were introduced before physical hazards [25]. Accordingly, in the chemical accident prevention system in Korea, studies on health hazards, such as acute toxicity, rather than explosive or flammable chemicals were actively conducted first. These regulations are applicable to some chemicals (hydrochloric acid, hydrofluoric acid, etc.) causing acute toxicity, however it is difficult to apply them to flammable and explosive chemicals. This study has pointed out the background and institutional limitations, and re-evaluated the designated quantity of chemicals in terms of chemical accidents.

### 3.2. Comparison of Damage Radius by Chemicals

The results of modeling of the chemical accident case for pool fires and VCEs using programs and materials are as follows. The damage radius expected for all chemical accidents is similar. Currently, there is a 20-fold difference in the designated quantity between benzene and xylene but the damage radius is similar. In particular, the modeling results under similar conditions showed similar damage radii, indicating that chemical accidents are highly related to the physicochemical properties of chemicals. Table 6 shows the damage radius for each modeling program. The similarities between the results of each program are also significant. For xylene, the result was that the VCE model was impossible in ALOHA. VCEs occur mainly indoors because steam remains for a long time and explodes as an ignition source, or in outdoor cases the atmospheric conditions are stagnant (without wind). It was concluded that in the case of xylene, it is difficult to generate a VCE model because no vapor cloud is formed at the lower explosion limit.

**Figure 2:**
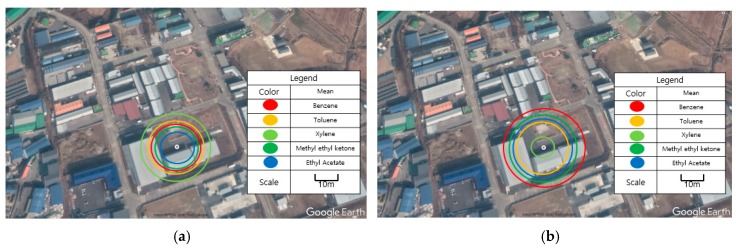
Damage radius of a (**a**) pool fire and (**b**) VCE.

Each damage radius for each modeling program is similar if the average value is applied. The range of effects are displayed at a glance in Figure 2. In the case of pool fires and VCEs, the damage radius for high-damage materials is different. However, overall, the chemicals show similar influence ranges. For the xylene VCE case, only KORA results were used. This is because a VCE event may not occur under the current conditions, however an accident may occur when a leaked pool becomes large, an indoor storage tank is considered, or when the atmosphere is congested. These types of accidents are more affected by VCEs than by pool fires.

### 3.3. Comparison and Proposal of Designated Quantity in Accident Prevention System

Based on the results of this study, we propose designated quantity values for the accident prevention system. Currently, Korea’s designated quantities for the accident prevention system differ greatly by chemical substance compared with the US and EU. This appears to be because Korea’s designated quantity values are made by considering the physical and chemical properties, the toxicity level, and the amount of domestic consumption. However, as mentioned above, in order to prevent accidents involving flammable substances, the designated quantity values should be calculated in consideration of the worst case, where a fire or explosion may occur.

If the same flammable substance is used but benzene is handled by a plant with 10 m^3^ tanks, the system is subject to safety regulations. However, a plant dealing with xylene is only subject to an accident prevention system if it has a 200 m^3^ tank. There is also a problem regarding equity. In addition, in the event of a chemical accident, these results may affect the workplace or neighboring residents.

According to the previous research, the ration of pool-fire-to-VCE occurrence was 7:3 [26]. The designated quantity was calculated based on the minimum designated quantity of benzene (10,000 kg). The larger the range of influence, the more dangerous the substance, and therefore the smaller the quantity specified. Since benzene had the greatest overall damage radius, the product of the total damage radius and the specified quantity divided by the total range of influence of other study materials was presented as the designated quantity (Table 7).

The designated quantity suggested by the US and EU is 10,000 kg for all chemicals classified int the flammable group. This result does not deviate much from the designated quantity suggested by the research results. On the other hand, when comparing Korean designated quantities, there is a big difference. The quantity limits for toluene and methyl ethyl ketone were about 17 times greater, xylene was 15 times greater, while ethyl acetate was about 1.5 times greater.

## 4. Discussion

This study is a feasibility evaluation of the designated quantities for the chemical accident prevention system currently employed in Korea. Korea introduced the chemical accident prevention system used in advanced countries, such as the US and the EU, and applied it in accordance with the Korean situation. In particular, by benchmarking ALOHA, a modeling program used by the US EPA, many workplaces are striving to prevent and reduce chemical accidents by developing and applying KORA, a modeling program that is applicable in Korea.

However, there are limitations on designated quantities in the process of the comprehensive application of various aspects, for example physicochemical properties, inherent toxicity, and domestic consumption.

In the US and the EU, chemicals with flashpoints below 60 °C are grouped and managed rather than subdivided into chemicals. Unlike some unique chemicals, such as hydrochloric acid or sulfuric acid, which have large differences in vapor pressure, flammable chemicals have similar physicochemical properties. Of course, in the US, some substances with flash points below 60 °C require some control, for which separate criteria are provided (e.g., toluene diisocyanate).

According to the research results, the designated quantity in Korea is somewhat underestimated. Assessing chemical accidents with designated quantities seems to suggest that toluene is 20 times safer than benzene. Chemical accidents require a preventive and conservative approach. Simulation of fires and explosions that could cause chemical accidents based on benzene, which among the flammable substance group has the most conservative designated quantity, showed that the proper designated quantity was between 10,000 and 13,500 kg. This causes a difference of up to 17-fold from the current designated quantities.

According to the modeling conditions, it was also found that xylene is problematic for VCE modeling [27]. Under the same conditions, xylene is unlikely to cause an accident, but it has the same quantity limit as toluene and methyl ethyl ketone. In fact, considering the overall range of impacts, ethyl acetate is less dangerous than methyl ethyl ketone.

In Korea’s Chemicals Control Act, 97 kinds of “chemicals requiring preparation for accidents” are designated as of 2020. In addition to the five substances studied, 97 substances are composed of chemicals, such as sulfuric acid, hydrochloric acid, and nitric acid, which are highly likely to cause chemical accidents and are likely to cause harm to the human body. The advantage is that it is easy to manage and recognize handling of chemicals in the workplace. In contrast, employers will not implement an accident prevention management system if they only operate below the designated quantity [5].

Methyl ethyl ketone and other flammable chemicals are ranked differently in terms of both fire and VCE risk. However, the main conclusion of this study is that the damage radii of chemicals showed similar results. The modeling programs ALOHA and KORA are likely to differ due to the nature of Korea as a developing country. For example, KORA was developed in Korea, so some of the toxic properties may have been applied to the modeling conditions. However, the results of Table 7, showing a feasibility evaluation of specified quantities for chemicals, show that the designated quantities of all chemicals do not decrease or increase by more than 40% based on benzene. This is a statutory regulation, since it is in excess of 10,000 kg, the designated quantity for flammable substances in the US and EU. However, in Korea, the designated quantities are overestimated by up to 2000%.

According to the results of this study, it is necessary to improve the system by grouping 97 similar physicochemical properties of “chemicals requiring preparation for accidents”. Alternatively, it is also necessary to reassign the designated quantity through modeling by approaching chemical accidents. As for the US or the EU, the Korean legislation needs to be revised, and other countries need a basic national management system to prevent chemical accidents.

## 5. Conclusions

This study is about the feasibility assessment of the designated quantities of chemicals that are subject to the Korean chemical accident prevention system. Unlike in the US and the EU, Korea’s reserves are underestimated. Chemical accidents need to be managed through a conservative approach. In particular, for flammable substances, unlike toxic substance leakage, accidents occur immediately without and initial response, meaning the damage is large. In addition, safety management is more important, in that the initial accident can cause domino explosions and fires several times, instead of just one accident [28]. Designated quantity is of great importance as a criterion for the minimum safety management system to be applied in the workplace. This is because workplaces working with less than the specified quantity are not legally required to implement safety management. Therefore, with the most conservative approach, many chemical handling sites should be required to implement the safety management system.

**Figure 3:**
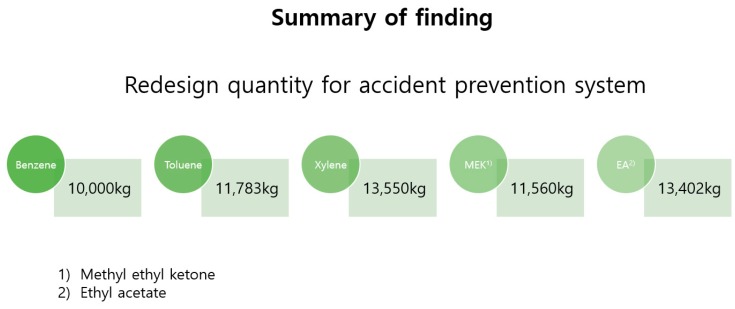
Summary of findings.

The US and the EU group all similar flammable substances together and suggest 10,000 kg for minimum quantities. However, in Korea, the quantities of flammable substances, such as benzene, toluene, xylene, methyl ethyl ketone, and ethyl acetate, differ from 10,000 to 200,000 kg. This could be seen as a blind spot in the law, depending on the substance handled (Figure 3).

For modeling of a fire or explosion, the quantity limit results were in the range of 10,000–13,500 kg, well below the current legally specified maximum quantity of 200,000 kg. This does simply with improve the system, but means that the possibility of chemical accidents should be considered as the biggest factor in the selection of designated quantities and that the safety system should be implemented.

## Figures and Tables

**Table 1:** Physicochemical properties of chemicals requiring preparation chemicals. RMP, risk management plan.

Representative Characteristics of Chemicals	Benzene	Methyl Ethyl Ketone	Toluene	Xylene	Ethyl Acetate
CAS (Chemical Abstracts Service) no.	71-43-2	78-93-3	108-88-3	1330-20-7	141-78-6
Molecular formula	C_6_H_6_	C_4_H_8_O	C_7_H_8_	C_8_H_10_	C_4_H_8_O_2_
Molecular weight	78.11	72.11	92.14	106.17	88.11
Flash point/ignition point	(−11/497) °C	(−5.6/404) °C	(−4.4/480) °C	(29/65) °C	(−4/427) °C
Upper explosion limit	7.8/1.2	11.5/1.8	7.1/1.1	7.0/1.1	11.5/2.2
Vapor pressure	94.8 mmHg (25 °C)	90.6 mmHg (25 °C)	28.4 mmHg (25 °C)	8.29 mmHg (25 °C)	93.2 mmHg (25 °C)
Acute toxicity (Lethal Dose 50; LD 50)	930 mg/kg mouse	2600 mg/kg mouse	5580 mg/kg mouse	3580 mg/kg mouse	5620 mg/kg mouse
Quantity RMP	10,000 kg	200,000 kg	200,000 kg	200,000 kg	20,000 kg

**Table 2:** Comparison of designated quantity of accident prevention system of chemicals by country.

Country Name	Benzene	Methyl Ethyl Ketone	Toluene	Xylene	Ethyl Acetate
US	Flammable Liquids 10,000 kg ^(1) (2)^				
EU (UK)	Flammable Liquids 10,000 kg				
Korea	10,000 kg ^(1)^ 5000 kg ^(2)^	200,000 kg ^(1)^ 5000 kg ^(2)^	200,000 kg ^(1)^ 5000 kg ^(2)^	200,000 kg ^(1)^ 5000 kg ^(2)^	20,000 kg ^(1)^ 5000 kg ^(2)^

(1) RMP designated quantity. (2) Process safety management (PSM) designated quantity.

**Table 3:** Modeling conditions.

Storage Tank Material and Specifications					Operating Meteorological Conditions			
Material	Capacity (m^3^)		Dike Size	Distance from the Ground (m)	Average Temperature (°C)	Average Humidity (%)	Average Wind Speed (m/s)	The Meteorological Stability ^4^
	Design (mm)	Operation						
STS ^3^ 304	10(D ^1^: 2500; H ^2^: 2700)	10	Horizontal: 4500 Vertical: 4500 Area: 15.35 m^2^ Volume: 16.85 m^3^	0.1	25	1.5	1.5	D ^4^

^1^ D: diameter; ^2^ H: height; ^3^ STS 304: stainless steel material; ^4^ meteorological stability for Pasquill’s classes.

**Table 4:** Meteorological stability for Pasquill’s classes.

Wind Speed (m/s)	Day-Time			Night-Time	
	Size of Radiation Intensity				
	Strong	Moderate	Slight	Cloudy	Sunny
<2	A	A–B	B	F	F
2–3	A–B	B	C	E	F
3–5	B	B–C	C	D	E
5–6	C	C–D	D	D	D
6>	C	D	D	D	D

A: very unstable; B: instability; C: slight instability; D: neutral; E: slightly stable; F: very stable.

**Table 5:** Characteristics, advantages, and disadvantages of risk assessment tools. ALOHA, Areal Location of Hazardous Atmosphere; KORA, Korea Off-Site Risk Assessment Supporting Tool.

Characteristics by Modeling Program	ALOHA	KORA
Pool fire model	Available, leak time and leak area can be input	Available, leak time and leak area can be input
VCE model	Baker–Strehlow–Tang (BST) method	The Netherlands Organization (TNO) multi-energy method
Pipe leak model	Available	Not available
Major Usage	Derives a simple result for a variety of uses, initial factory location analysis	For compliance with Korean regulations. When establishing an RMP, determines the scope of communication with local residents
Advantages	Free (US EPA), provides quick results, applicable to the US and Korean PSM and RMP	Free (Korea Ministry of Environment), provides quick results, applicable only to Korean PSM and RMP
Disadvantages	Impossible to calculate three-dimensional concentration distribution, impossible to realize atmospheric chemical reaction, prediction to 10 m	Impossible to calculate three-dimensional concentration distribution, does not support piping leak models

**Table 6:** Comparison of damage radius by modeling program.

Chemical	ALOHA		KORA	
	Pool Fire Accident Damage Radius (m)	VCE Accident Damage Radius (m)	Pool Fire Accident Damage Radius (m)	VCE Accident Damage Radius (m)
Benzene	14	20	10.4	16.7
Toluene	14	11	10.5	11.3
Xylene	13	No part of the cloud is above the LEL at any time	13.4	7.5
Methyl ethyl ketone	13	15	8.9	14.9
Ethyl acetate	10	14	7.9	14.1

LEL: low explosion limit.

**Table 7:** Feasibility evaluation of specified quantities for each chemical.

Chemical	Total Damage Radius (m)	Designated Quantity Suggested by the Research Results (kg)	Current Designated Quantity (kg)
Benzene	14.04	10,000	10,000
Toluene	11.92	11,783	200,000
Xylene	10.37	13,550	200,000
Methyl ethyl ketone	12.15	11,560	200,000
Ethyl acetate	10.48	13,402	20,000
